# The Human Oral Microbiome in Health and Disease: From Sequences to Ecosystems

**DOI:** 10.3390/microorganisms8020308

**Published:** 2020-02-23

**Authors:** Jesse R. Willis, Toni Gabaldón

**Affiliations:** 1 Barcelona Supercomputing Centre (BCS-CNS), Jordi Girona, 29., 08034 Barcelona, Spain; 2 Institute for Research in Biomedicine (IRB), The Barcelona Institute of Science and Technology (BIST), 08034 Barcelona, Spain; 3 Institució Catalana de Recerca i Estudis Avançats (ICREA), Pg. Lluís Companys 23, 08010 Barcelona, Spain

**Keywords:** Oral microbiome, Next generation sequencing, oral diseases, systemic diseases, stomatotypes, microbiome perturbations

## Abstract

The human oral cavity is home to an abundant and diverse microbial community (i.e., the oral microbiome), whose composition and roles in health and disease have been the focus of intense research in recent years. Thanks to developments in sequencing-based approaches, such as 16S ribosomal RNA metabarcoding, whole metagenome shotgun sequencing, or meta-transcriptomics, we now can efficiently explore the diversity and roles of oral microbes, even if unculturable. Recent sequencing-based studies have charted oral ecosystems and how they change due to lifestyle or disease conditions. As studies progress, there is increasing evidence of an important role of the oral microbiome in diverse health conditions, which are not limited to diseases of the oral cavity. This, in turn, opens new avenues for microbiome-based diagnostics and therapeutics that benefit from the easy accessibility of the oral cavity for microbiome monitoring and manipulation. Yet, many challenges remain ahead. In this review, we survey the main sequencing-based methodologies that are currently used to explore the oral microbiome and highlight major findings enabled by these approaches. Finally, we discuss future prospects in the field.

## 1. Introduction

Much like the various terrestrial biomes that make up the Earth, the human microbiome is a series of distinct communities of bacteria, fungi, viruses, archaea, protists, and other microorganisms, whose compositions are dependent upon environmental conditions [1]. Different sites of the human body can be seen as unique biomes, with drastically different environments and nutrient availabilities, which in turn promote different communities. Yet even within a particular body site, the microbiome composition can be highly variable between individuals in different states of health, with distinct lifestyles, or due to a number of other factors [2]. The focus of this review will be the human oral microbiome, techniques to approaching its analysis, and outlining its typical composition as we currently know it, as well as its deviations under atypical conditions. 

The oral cavity contains one of the most diverse and unique communities of microbes in the human body [3,4], yet this niche is relatively understudied as compared to the gut—at the time of writing this review, a PubMed search with “oral microbiome” resulted in 746 articles, as compared to 5605 with “gut microbiome”. A milliliter of saliva contains approximately 10^8^ microbial cells [5], and an array of studies have detected up to 700 distinct prokaryotic taxa [6], with a typical healthy microbiome comprised of a range of about 100 to 200 distinct bacterial organisms [7]. The advent of next generation sequencing (NGS) techniques has opened new avenues for large-scale metagenomic studies in diverse populations, allowing for characterization of the microbiome structure and, in some cases, the functional roles and implications for health.

The mouth as a biome is home to multiple unique habitats, each of which has its own community of microorganisms. The microbiomes of the saliva, tongue, buccal mucosa, teeth surfaces, gums, palate, both subgingival and supragingival plaque, as well as the throat and tonsils, have all been characterized in multiple studies, showing overall similarities but with small-scale differences, such as higher levels of the genus *Corynebacterium* in both types of plaque [3,8] or higher levels of the phylum Firmicutes in both saliva and buccal mucosa as compared to plaque [8,9]. While some metagenomics studies look at these individual habitats separately, it is also not uncommon to use an oral rinse as a sample collection method, in order to obtain a representative sample of the overall oral microbiome [10,11,12]. 

Regardless of the particular biome or habitat being explored, the current trend in microbiome studies is largely in taking advantage of culture-independent NGS technologies, as they continue to decrease in both financial and computational cost, alongside the continuous expansions of databases of microbial genetic sequences. According to the expanded Human Oral Microbiome Database (HOMD) [13], only 57% of the oral bacterial species have been officially named, 13% have been cultivated yet remain unnamed, and 30% are uncultivated. Hence, not only do the NGS techniques make analyses relatively quick and easy, but they have also vastly expanded our awareness of unculturable and/or rare microbiota.

The mouth can be affected by several pathologies that have high prevalence among human populations, including periodontitis, gingivitis, and dental caries, all of which have been clearly related to alterations in the oral microbiome (see references in Table 1). However, the mouth constitutes an entry point to the respiratory and digestive systems, and it is highly vascularized, resulting in potential implications of the oral microbiome in other systemic diseases. Indeed, a growing number of studies have shown associations between other diseases and changes in the oral microbiome (Table 2). This suggests that oral microbiota may provide potential biomarkers in the diagnosis of some systemic diseases.

As we search for these deviations within populations, we must also consider the caveat that our knowledge of the human microbiome may be far from complete. Recent studies have collected data from previously unstudied populations and found not only differences in composition but even added many undiscovered species to public databases [44,45], highlighting strong disparities between different regions of the world. Most studies have been focused on European, North American, Chinese, or other so-called “WEIRD” populations. This refers to Western, educated, industrialized, rich, and democratic nations, an acronym that was proposed originally to denote a bias in psychology studies toward these societies, which at the time comprised about 13% of the world’s population, yet accounted for between 60% and 90% of subjects in psychology studies [46]. This early evidence of a similar bias in microbiome studies suggests that there remains the possibility of a much broader landscape of “healthy” microbiomes across different cultures. Furthermore, even within healthy sample sets of the same populations, distinct subgroups can be elucidated [10,47,48,49,50,51], Therefore, in addition to a focus on understanding the causes and effects of dysbiosis in the microbiome, there must also be a continued emphasis on fully characterizing the healthy microbiome to more reliably detect true deviations from the normal state.

Studies aiming to characterize the composition of the oral microbiome in diverse human populations are progressing rapidly, as are those looking for variation according to a growing number of parameters, particularly those related to health and disease. In this review, we provide a general overview of the state-of-the-art methodologies used to study the oral microbiome, and of the main results obtained during the last decade of intense research. Finally, we will discuss the current challenges and perspectives of this fast-moving field. Throughout the review, we will put a focus on the emerging roles of the oral microbiome in health and disease, and the new opportunities for therapeutics and diagnostics.

## 2. Technical Approaches to Study the Oral Microbiome

**Figure 1:**
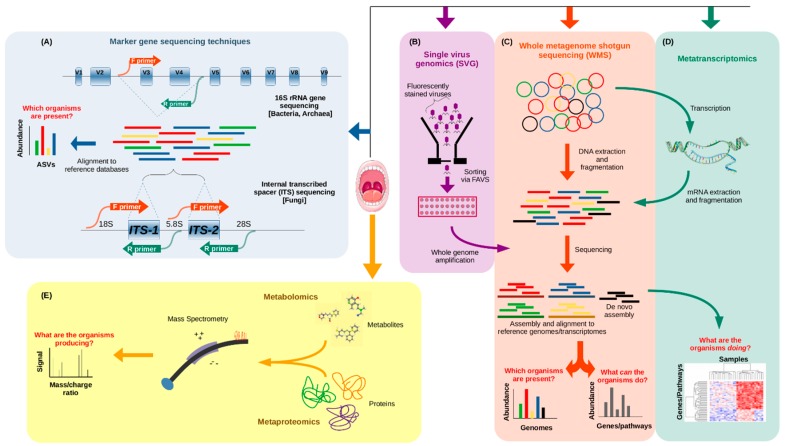
Schematics of standard techniques used in microbiome studies. (**A**) Marker gene sequencing techniques can use primers to target certain conserved regions of a genome to capture intermittent variable regions, which can then be used to identify organisms in a sample rapidly and inexpensively. The 16S rRNA gene is the most commonly used marker gene in bacteria and archaea, and in the figure, primers are used to capture the V3 and V4 variable regions together, a common approach for 16S sequencing. The internal transcribed spacer (ITS) region of the nuclear rRNA cistron in fungi is made of two segments, which can be captured with primers targeting the 18S, 5.8S, and 28S rRNA sections that surround them. (**B**–**D**) Instead of targeting one small segment of the genome, these techniques capture the entirety of the genetic material from an organism. (**B**) Single virus genomics (SVG) uses a fluorescent stain to isolate individual virus particles in a sample by fluorescence-activated virus sorting (FAVS), wherein they are embedded in an agarose bead before undergoing whole genome amplification and sequencing. (**C**) Whole metagenome shotgun sequencing (WMS) involves the fragmentation of all DNA in a sample, sequencing of the fragments, and assembly of the sequences, which can then be mapped to reference genomes, or de novo assembly can be performed. (**D**) Metatranscriptomics also involves a shotgun sequencing approach, but it is performed after mRNA extraction. The outputs then allow for differential gene expression analysis. (**E**) Metabolomics and metaproteomics allow for quantification of the metabolites and proteins produced by the microbiome in a sample, respectively. Mass spectrometry is a common approach to quantification. Mock metabolite shapes in Figure 1 were generated using the JSME Molecular Editor by Peter Ertl and Bruno Bienfait licensed under CC-BY-NC-SA 3.0. Images of body sites and organs in Figure 1 and Figure 2 were obtained from Servier Medical Art by Servier licensed under CC-BY 3.0.Traditionally, 16S sequences were clustered into groups with at least 97% identity, called operational taxonomic units (OTUs), which have been used as proxies for species-level or, more commonly, genus-level taxonomic identification. A number of software tools are available, which convert reads to sample-by-OTU feature tables, such as QIIME [55] and mothur [56]. However, newer approaches are better able to control for amplicon sequencing errors, and thereby obviate the use of arbitrary identity thresholds, allowing for single-nucleotide resolution with amplicon sequence variants (ASVs) [57]. Software options for ASV methods include DADA2 [58] and Deblur [59].

The costs of sequencing DNA have plummeted thanks to the introduction of NGS technologies, now allowing scientists to sequence several human genomes in a single day at a price of under $1000 per genome, a nearly one million-fold decrease from 20 years ago [52]. Similarly, there are a number of cost-efficient NGS techniques that can be used today when approaching microbiome studies, depending on what the researcher hopes to learn (Figure 1). The two most widely used approaches include whole metagenome shotgun sequencing (WMS) and 16S ribosomal RNA amplicon sequencing, both of which involve reading the DNA sequences of the microbes present in a sample and comparing them to a database of sequences to establish the relative quantities of the different organisms present in that sample. In WMS sequencing (Figure 1C), the DNA is randomly fragmented multiple times, allowing millions of short sequences to be read in parallel, and then they are reassembled into full (or partial) genomic sequences by connecting the overlapping ends [53]. However, 16S rRNA sequencing (Figure 1A), also known as 16S barcoding, has been used more frequently in metagenomic studies, since it is less expensive, both experimentally and computationally, permitting larger scale study designs. The 16S rRNA gene is common to all bacteria and archaea, and has highly conserved regions, which make it a useful marker gene for the use of universal primer sequences to isolate it for sequencing. Scattered amongst the conserved regions of the gene are nine hypervariable regions (named V1 through to V9), and it is these segments that allow the taxonomic identification of organisms when mapping reads to a database of known 16S rRNA sequences [54].

Of course, even with the use of ASVs for identification, 16S sequencing still lacks significant taxonomic resolution as compared to WMS sequencing, often only permitting distinction up to the genus level. Alternatives to 16S sequencing have also been proposed in order to improve resolution or to avoid bias due to the varying number of copies of the 16S gene in different species [60] (though there are methods to correct for it [61]). The *rpoB* gene, for instance, has the advantage of generally being single copy and having greater variation, which allows for deeper taxonomic resolution. However, the corresponding lack of conservation makes it less applicable as a universal marker [62]. A database of *rpoB* gene sequences is available from the FROGS (Find, Rapidly, Otus with Galaxy Solution) website [63]. Some have proposed that one or more housekeeping genes, like *rpoB*, should be sequenced along with the 16S gene, since they are ubiquitous and rapidly evolving, allowing for better taxonomic resolution than the 16S gene alone [64]. To distinguish closely related organisms, others have suggested a multilocus sequence analysis (MLSA) approach, wherein multiple housekeeping genes from distinct chromosomal loci are sequenced in parallel [65]. Nevertheless, the 16S rRNA gene remains the current standard for marker gene analyses of the microbiome.

All of the marker gene techniques mentioned are useful when asking the question, “What microorganisms are present in a sample?”, giving an overview of the microbial makeup across many samples. However, WMS sequencing can allow for the detection of species or even strains, in addition to functional annotations of microbiome samples [66], which can only be predicted based on known full genome sequences when performing 16S sequencing. So, WMS additionally gives insight into the functional potential of the microbiome, allowing researchers to ask the question “What can the microorganisms present actually do?”.

Metagenome studies can be further bolstered by the use of metatranscriptomics [67], metaproteomics [68], and metabolomics [69], though only the first of these utilizes NGS technologies. Metatranscriptomics (Figure 1D) answers the question, “what are the microorganisms doing?” Here, the idea is to profile the total microbial gene expression in a sample by capturing the total messenger RNA (mRNA) content, so this is particularly useful when exploring the functional activity of the microbiome in different conditions, like disease vs. health, different diets, or different times of the day. Metaproteomics (Figure 1E) is another approach to assessing the functional activity of a microbiome sample, but instead of sequencing genetic material, the idea is to catalog the abundances of the microbial proteins present in a sample. This is typically done by protein extraction and tandem mass spectrometry analysis (MS/MS) [68]. Metabolomics (Figure 1E), on the other hand, answers the question, “what are the microorganisms producing in a given sample?” The metabolome is the total set of small molecules produced by the microbiome (and the host) in a sample, and can be a strong indicator of the health or dysbiosis of a sample [70]. Metabolites are typically quantified by use of chromatography and detection techniques like mass spectrometry (MS) and nuclear magnetic resonance (NMR). Each of these techniques also has its own drawbacks, which prevent it from being as widely used as metagenomics. Metatranscriptomics can be hindered by the instability of mRNA and the excess of rRNA (though methods have been developed to counteract this [71]), and by the limited reference databases of transcriptomes [72]. Metaproteomics suffers from computational limitations when querying protein databases (which, nevertheless, remain incomplete), as well as a redundancy in annotations due to identical peptides in homologous proteins from different organisms, which may use them in different processes, thereby leaving the resulting taxonomic and functional quantifications ambiguous [68]. Recent tools, however, claim to combat both of these issues [73]. With metabolomics, the challenges lie in determining whether the metabolites are produced by the host or the microbiome, and associating them with the relevant genes and pathways, highlighting the need for the integration of this technique with other omics data [72].

All of these tools and techniques are frequently aimed at investigating the bacteriome, which makes up the most significant portion of the microbiome but not its entirety. To classify the composition of the mycobiome, the fungal component of the microbiome, researchers often use a marker region, much like the 16S rRNA gene, called the internal transcribed spacer region of the nuclear ribosomal RNA cistron, referred to as the ITS region (Figure 1A), which provides a similar taxonomic resolution to that of 16S sequencing for bacteria [74]. The virome, the viral component of the microbiome, can be difficult to approach since there are no conserved marker regions like in the 16S rRNA gene in bacteria or the ITS region in fungi. Thus, the full virome must be sampled and compared to known viral sequences. Two problems arise from this, as current viral databases lack the characterization of many viruses, and consequently, any new viral sequences that do not match closely to those in current databases would be difficult to classify [75]. Another challenge for virome studies is the relatively low proportion of viral nucleic acid content alongside that of other microbes. However, there have been enrichment procedures proposed to increase the content of viral nucleic acids [76]. More recently, a new approach called single virus genomics (SVG) has been proposed (Figure 1B), in which individual viruses are isolated via fluorescence-activated viral sorting (FAVS), and genomic material is amplified and sequenced [77].

Whatever the technique being employed, it is important that researchers come to a consensus on the exact procedure for collection and sequencing to ensure reproducibility. While some of these referenced studies have shown that the microbiome profile of a sample is not heavily influenced by the collection technique [78,79], these are focused on large-scale differences. However, as sequencing technologies become more efficient and microbiome-associated databases become more complete, researchers will continue to compare samples at finer scales, so minor technical variability stemming from different techniques of swabbing collection sites or using a different solution for oral rinse collections could potentially impact results. Some reviews of the current best practices have been published [80,81] and these should continue to be improved and built upon.

Depending on the investigator’s study goals, there is a wide variety of potential approaches to data analysis, and there are equally plentiful software packages available. The phyloseq [82] and microbiome [83] packages for R offer a means to organize the data from sequencing experiments alongside any metadata, and provide a collection of tools and tutorials for calculations and plotting in typical microbiome analyses. This includes functions for calculating the alpha diversity (the relative diversity of taxa present in a sample) and beta diversity (the relative distance between any two samples based on the overall composition, as well as plots for various ordination methods. From there, a bioinformatician can go in any number of directions, so here we will just mention some of the most prominent analyses. The vegan R package [84] offers options for multivariate tests like the anosim function (analysis of similarities) to determine differences in microbiome compositions between groups of samples, and the adonis function for permanova (permutational multivariate analysis of variance), which can apply linear models to determine sources of variation amongst samples. Linear mixed effects models can also be applied to determine the effects on various data points, like particular taxa, diversity levels, or other metadata variables. Standard or generalized linear models can be fitted using the lm or glm functions, respectively, from the core R package called stats [85]. Mixed effects models can also be used with the lmer and glmer functions from the lme4 package [86]. There are also software options available to predict the functional capability of a microbiome sample sequenced with a marker gene target like 16S rRNA. The tools PICRUSt (phylogenetic investigation of communities by reconstruction of unobserved states) [87] and Tax4Fun [88] use reference genome databases to attempt to reconstruct full metagenomes from each sample. Machine learning techniques have also been implemented to attempt to predict disease based on the microbiome composition [89], and some investigators have made their code publicly available [90,91]. The oral microbiome has even been used in a classifier for colorectal cancer with some success [92].

A recent point of contention in the field is that of the compositional nature of microbiome data and the implication for its analyses, something which some have begun to address from both an experimental [93] and a statistical [94] perspective. Since the reads produced in an NGS experiment are essentially random samples of the relative abundances of the organisms present, this cannot account for the implications of differences in the total abundances of organisms, which may be physiologically relevant. Vandeputte et al. described an experimental technique called quantitative microbiome profiling (QMP) [93] in which the total microbial load is determined by sampling an equal number of sequences per sample, and then correcting for the 16S copy number bias and the total number of cells from the sample. They were able to use this approach to reveal erroneous results from a standard experiment, suggesting a solution for some technical biases inherent in NGS studies. However, since most datasets to date have not been sequenced with any techniques like QMP, other solutions have been proposed to handle the typical asymmetrical datasets in microbiome studies. Rarefaction of read counts, in which a random sample of the same number of reads is extracted from each sample, was a common approach in earlier microbiome studies, but this practice has been discouraged as it omits biologically relevant information [95]. Gloor et al. instead suggested normalizing the data using a centered log-ratio transformation, which is minimally affected by the depth of reads for a sample [94]. They also provided a tutorial for the workflow that accompanies their publication on this topic.

## 3. The Oral Cavity and its Microbial Niches

**Figure 2:**
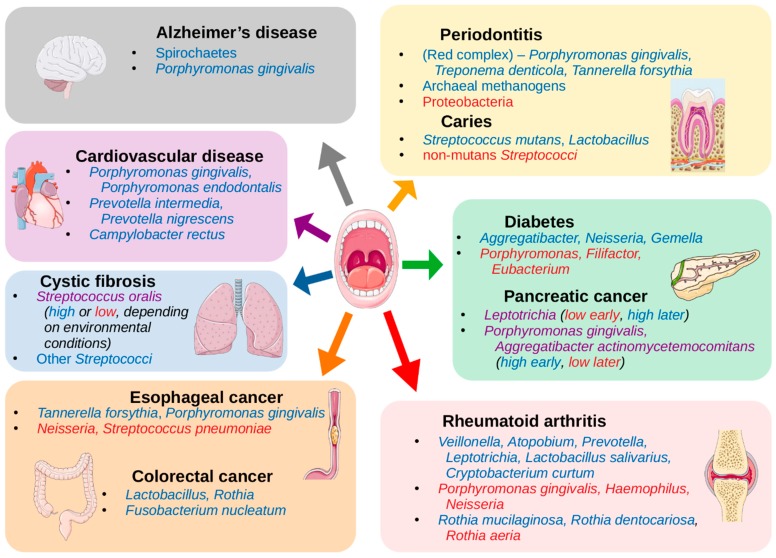
Oral and systemic diseases associated with the oral microbiome. A representation of the associations found between diseases with increases or decreases of the abundances of organisms in the oral cavity (listed in Table 1 and Table 2). Organisms listed in blue have been shown to be increased in abundance in the oral cavity in individuals presenting with the noted disease, and organisms listed in red have been shown to be decreased. Those in purple may be either increased or decreased depending on the conditions or progression of the disease. Images of body sites and organs in Figure 1 and Figure 2 were obtained from Servier Medical Art by Servier licensed under CC-BY 3.0.

Of all the habitats within the human body for which the microbiome is typically studied, the oral cavity warrants perhaps the most unique approach to study, in that it contains a number of highly distinct niches formed at the various surfaces within the mouth. Changes in the availability of oxygen, nutrients, and the pH-mediating effect of saliva [96] can promote the growth of different organisms, and conversely, these organisms can be involved in their own small niche construction [97] via biofilm formation and nutrient metabolism, which can produce effects both within the oral cavity (Table 1, Figure 2) and systemically (Table 2, Figure 2). Some researchers have chosen to study all these niches in parallel to compare them against each other [3,8,9], some have selected individual sites with particular focuses on localized dysbiosis in disease states [98,99,100,101,102,103,104,105,106], and others have used an oral rinse approach to capture an overall view of the oral cavity [10,11,12].

Studies focusing on specific oral niches typically aim to explore a disease relevant to that site. For instance, primary Sjögren’s syndrome (pSS) in the buccal mucosa was believed to be a potential reservoir for pathogens implicit in the disease, wherein the disease samples were shown to have higher Firmicutes/Proteobacteria ratios as compared to healthy controls, and higher abundances of 19 genera [98]. Various efforts have characterized the changes in subgingival and supragingival plaque related to periodontitis [99,100,101,102], as well as a study of subgingival plaque and buccal mucosa showing that both sites differed between periodontitis samples and healthy controls, with many of the same organisms affected in both sites, though they also displayed unique species colonization [103]. The tongue microbiome was explored in the elderly in Japan because of a potential connection between ingested microbes and pneumonia, which found that samples with worse dental health were enriched in pneumonia-associated bacteria [104]. The tongue was also targeted as a potential segment in diagnostic tools that would perhaps incorporate the microbiomes of the full gastrointestinal tract to detect pancreatic cancer [105]. The palatine tonsils were explored in HIV-infected patients to better understand the oral and systemic complications of the disease, and it was shown that the bacteriome was indeed significantly altered in infected individuals, but the mycobiome was not [106].

At a broad scale, the microbial composition throughout the regions of the oral cavity is fairly consistent, making it easily distinguishable from the microbiomes of other human body habitats [3,107,108,109,110]. However, while the niches in the oral cavity are largely composed of the same organisms, some may be present in different proportions. One study combining samples from 10 niches along the digestive tract in over 200 individuals from the United States placed these niches into four groups based on the similarity of overall composition [8]. One of the sites was the intestine, represented by stool samples, which were grouped alone, while the other nine were in the mouth and throat. One of the three other groups consisted of buccal mucosa, keratinized gingiva, and hard palate, another of tongue, saliva, palatine tonsils, and throat, while the last group contained subgingival and supragingival plaques. Though all of the non-stool niches were generally dominated by the phyla Firmicutes and Bacteroidetes, they based the groups more on small-scale differences. The first group was more unique than the other two non-stool groups, and was shown to have a considerably higher abundances of the genus *Streptococcus* and lower overall alpha diversity, which is a measure of the relative diversity of organisms present in a given sample. Group 3, containing the two types of gingival plaque, typically had higher alpha diversity. Comparisons between the compositions of these niches and their diversities have been corroborated in other studies [3,9,111]. The authors posit that the level of saliva flow in the mouth is a key factor determining the composition of the microbiome at each niche in the oral habitat because of its capacity to regulate pH and nutrient availability, but other major factors may include the type of surface and oxygen availability. The two plaques, for instance, form on the non-shedding surfaces of teeth where they produce biofilms, within which oxygen is limited, resulting in greater abundances of obligate anaerobic organisms in the subgingival plaque and of facultative anaerobic organisms in the supragingival plaque [8]. They suggest that one niche from each group could be used to represent all of the niches in that group, such as using the buccal mucosa microbiome as a proxy for both keratinized gingiva and hard palate microbiomes (group 1 niches). However, as sequencing techniques continue to improve and costs continue to decrease, we may find more and more subtle idiosyncrasies within each niche, due to combinations of the microenvironmental factors just mentioned, as well as any others. So, that decision will be up to the discretion of the researchers and the relevance to their studies.

Since these niches tend to have very similar overall microbiome compositions at all but the lowest taxonomic levels, many researchers choose to treat the oral cavity as an individual habitat and analyze global compositions and processes therein [10,12,112]. A few studies have shown that this is a viable and efficient method for sample collection to investigate the oral microbiome [78,79], and standardized procedures have been proposed [113,114]. Essentially, an individual would refrain from eating, drinking, brushing, or smoking (anything that might temporarily shift the typical microbial composition) for at least 30 min prior to collection. Then, they would swish with a buffer solution for about 30−60 s, and then spit the contents into a tube, which would later be centrifuged and sequenced. The practical benefits of using an oral rinse are the ease of collection, as it is a quick and non-invasive method to obtain oral microbial DNA from a study participant, as well as the ease of storage and transport, since these samples can be frozen and sequenced later without detriment to the quality of the samples [79,115]. This is advantageous for large-scale microbiome projects, as it allows for the collection of many samples, which may take weeks or months, that can later be sequenced together to minimize the potential technical bias inherent in sequencing projects, as mentioned in the previous section.

## 4. The Healthy Oral Microbiome and Definition of Stomatotypes

The field of microbiome research is arguably still in its infancy, as evidenced by the continuing efforts to expand the databases on known microbial genomes, and to combat the bias toward “WEIRD” populations, as mentioned in the introduction [44,45,116]. These factors alone make it difficult to effectively define what constitutes a “healthy” oral microbiome. However, on top of that, the accumulation of studies over the last decade or so have shown that, even within particular populations, there can potentially be multiple distinct trends of microbiome composition amongst individuals in relative general health [10,48,49,50,51]. As such, while we may be eager to investigate the microbiome’s relationship to disease, it is vital that we also continue to further define microbiome compositions in health among different populations, and explore the causes of shifts within or between these populations.

Projects based on oral rinse samples are ideal for observing how the microbiome of the oral habitat as a whole is affected by external factors. Of course, the phrase “external factors” could cover a wide spectrum of variables, but some with clear connections to the mouth are the water we drink and the food we eat. In a cohort of 1319 samples from healthy adolescents in Spain analyzed by 16S rRNA sequencing, it was shown that differences in the ionic composition of public drinking water was associated with shifts in the overall composition of the oral microbiome [10]. Samples from regions with greater alkalinity and greater levels of ions, such as sulfate (SO4) and sodium (Na), had higher abundances of genera, such as *Porphyromonas* and *Flavobacterium*, while regions with lower levels showed higher abundances of other genera, including *Veillonella*, *Pseudomonas*, and *Ralstonia*. Different diets have also been shown to contribute to variations in the microbiome composition, such as in the WMS-based study comparing the oral microbiomes from populations of hunter-gatherers (HGs) from the Philippines, traditional farmers (TFs) from the Philippines, and Western controls (WCs) from the Human Microbiome Project (samples from the United States) [116]. They showed that the HG samples had higher alpha-diversity while it was lower in WC samples, and TF samples fell in the middle. Likewise, there was a strong gradient in the abundances of the core oral genera *Neisseria* and *Haemophilus*, with high levels of *Neisseria* and low levels of *Haemophilus* in HG samples, the reverse in WC samples, and TF samples again falling in between. The HG samples, despite good oral health, also displayed higher abundances of a number of species typically considered to be oral pathogens associated with gingivitis and periodontitis by Western standards. Functional analyses revealed an increase in vitamin B5 biosynthesis pathways in HG samples and, to a lesser extent, in TF samples. Americans had been shown to consume greater quantities of foods with vitamin B5, so the authors posit that this lack in hunter-gatherer diets would select for organisms that synthesize it on their own. Conversely, they showed that WC samples, and to a lesser extent, TF samples were enriched in urease activity, particularly from *Haemophilus* spp. This urease counteracts the drops in pH that occur when bacteria degrade sugars into acidic compounds, so selecting for these organisms in WC samples, with their sugar- and starch-heavy diets makes sense. The authors thus suggest that organisms considered to be oral pathogens in Western populations could indeed be part of the healthy microbiomes of different populations like hunter-gatherer societies, and that pathogenic strains of these organisms would be selected based on the nutrient availability tied to diet.

Food and water are obvious influencing factors, but any number of other factors could also impact the microbiome. A common approach in the early stages of the analysis of the microbiome of any body habitat is to first look at the broad effects of such factors by clustering the samples based on the overall microbial composition. The notion of separating samples into clusters was discussed in an early NGS-based study on the gut microbiome, wherein the authors labeled the clusters “enterotypes”, implying the presence of different putative categories of gut microbial composition [47]. They found three distinct enterotypes, the separations of which were driven largely by differences in the abundances of particular organisms, namely the genera *Bacteroides*, *Prevotella*, and *Ruminococcus*, and corroborated these by finding very similar enterotypes from two separate sample sets. From this, they suggested that there may exist some limited number of equilibria of symbiotic states between a human host and its microbiome, which would arise due to different diets and lifestyles.

Many studies have since adopted this technique, and in studies of the oral cavity, similarly composed clusters of samples have also emerged, dubbed “stomatotypes” in one such study, as homage to the original term enterotype, but in reference to the mouth [10]. A summary of some of the genera of bacteria that have been found to co-occur in different stomatotypes across studies is found in Table 3. There have thus been shown at least two strongly corroborated stomatotypes, one including higher abundances of the Proteobacteria genera *Neisseria* and *Haemophilus*, and the other with higher abundances of the Bacteroidetes genus *Prevotella* and the Firmicutes genus *Veillonella*. Some studies have shown more than just these two stomatotypes, though the consensus of compositions is more varied. Some of the genera co-occur in different manners, depending on the study.

**Figure 3:**
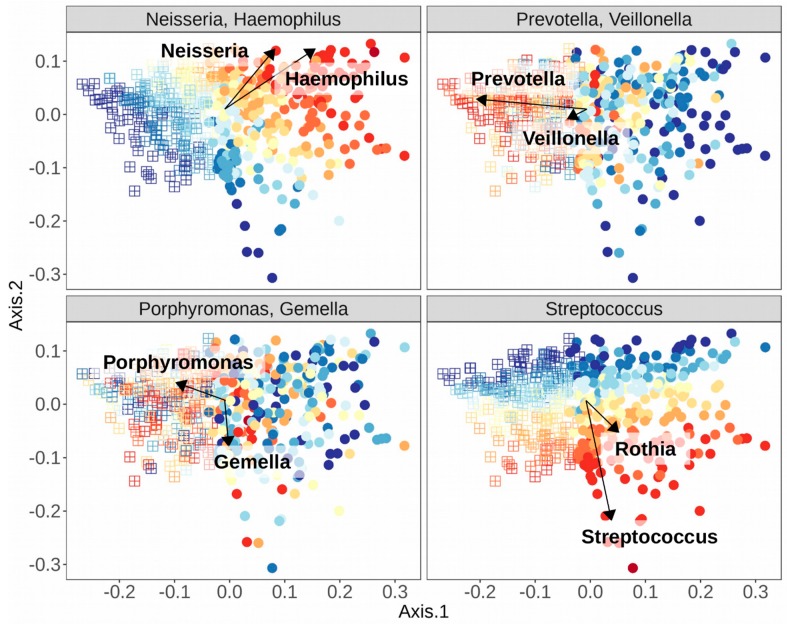
Gradients of abundances of consensus stomatotype-driving genera. Using a random subset of 500 samples from an oral microbiome dataset [10], samples were clustered into two stomatotypes using the weighted Unifrac distance measure. Type 1 samples are represented by circles and type 2 samples by squares. In each box, samples are colored by the total relative abundance of the indicated organisms. Overlaid are arrows indicating the tendency of the abundances of each organism noted in Table 3. In this subset of samples, *Neisseria* and *Haemophilus* strongly associate with stomatotype 1 samples, *Prevotella* strongly associates with stomatotype 2 samples while *Veillonella* does so weakly. The “variable stomatotype” drivers are indeed variable in their associations in this instance. *Streptococcus* shows a clear gradient but does not conform to either stomatotype. *Gemella* and *Rothia*, which have been shown to co-occur with *Streptococcus* in stomatotypes in the literature, do the same here, with *Rothia* more associated with stomatotype 1. However, *Porphyromonas*, which has been shown to co-occur with *Streptococcus, Gemella*, or *Neisseria* previously, associates with none of these here, and instead is strongly associated with stomatotype 2.

The explicit nature of stomatotypes may be very appealing as a means of differentiating samples, but these discrepancies highlight two major concerns. The first stems from the fragmentary nature of our current understanding of the microbiome due to the cultural biases and technical limitations already discussed. We do not yet have a complete picture of what may constitute the various potential equilibria of microbial abundances that lead to a particular stomatotype, because we have not explored the healthy microbiomes of many unique populations across the world, and many of the studies we already have lack the resolution to explain fine-scale distinctions between samples. As mentioned above, the hunter-gatherer populations from the Philippines that were sampled were enriched in *Neisseria* spp. while the Western controls were enriched in *Haemophilus* spp. [116]. This might suggest that samples from both populations would be clustered into stomatotype 1 from Table 3, since these two genera drive the equilibria of stomatotype 1, typically together but not in all studies [49]. Meanwhile, Bacteroidetes (the phylum containing *Prevotella*) and *Veillonella*, the drivers of stomatotype 2, fell more in the middle of the gradient between the HG and WC equilibria, further potential evidence that HG and WC may group together in stomatotype 1. How could one reconcile the strong differences displayed between these two populations and the evidence so far presented for the compositions within stomatotypes? This could likely be partially explained by the “WEIRD” bias in the studies presenting stomatotypes—perhaps these equilibria emerge in populations with westernized diets, medical treatments, and lifestyles. However, the question also ties into the second major concern, which is the statistical relevance of separating samples into discrete clusters. The notion of analyzing the gradients of microbial abundances was proposed as a response to the enterotype concept [117]. This has led to further discussions of the merits of enterotypes/stomatotypes, cautioning their use as predictive or diagnostic tools [118], and also suggesting improvements to their calculations while further emphasizing a focus on the gradients of abundance [107,119]. See Figure 3 for an example of the different gradients of the abundances of organisms in Table 3, which have been shown as stomatotype drivers, and how they associate with the stomatotypes found in a random subset of 500 samples from an oral microbiome dataset [10]. These, and other studies, suggest that stomatotypes are useful as a first step in exploring underlying variation among samples, which can then be further investigated through deeper analysis of shifts in particular organisms. For instance, in the study of adolescents in Spain, they drew connections to tap water composition by first observing maps of the distributions of samples in the two stomatotypes that they found, which were reminiscent of maps of water hardness values across Spain, and then later began to look at the effects on particular organisms within their samples [10].

## 5. Non-Bacterial Oral Microbes

Bacteria dominate both the research about the human oral microbiome, and the biomass within the oral habitat, with fungi estimated to comprise <0.1% [120]. Nevertheless, there is an appreciable diversity of fungal species present in the oral cavity, including species from the genera *Candida*, *Aspergillus*, *Penicillium*, *Schizophyllum*, *Rhodotorula*, and *Gibberella* [121]. Yet, two primary complications have limited the exploration of the mycobiome: (1) Difficulty in identifying many fungal species and (2) confusion in fungal nomenclature. Both of these issues have begun to be addressed in large part by the use of NGS technologies. Until recently, the diversity within the oral mycobiome was believed to be quite limited, dominated primarily by a few species of *Candida* [122]. This was largely because many fungi are difficult to cultivate in a laboratory, but advances in NGS technologies have revealed a wider array of fungal organisms than previously expected. One study found that the genus *Malassezia* was highly prevalent in the mouth [123] but had previously gone undetected in this body site because it has particular lipid requirements and needs specialized culture media to grow in a lab, and it was previously believed to be a pathogen on the skin [124]. However, even within metagenomic studies, there may be complications in categorizing the true fungal diversity. For instance, a study re-analyzing the samples in which *Malassezia* was detected was not able to find this genus in any of the samples [125], but it is possibly because this second study did not use the same DNA extraction protocol as the first, which included a step that used beads to help break cell walls and capsules. This highlights an inconsistency in the protocols for fungal metagenomic studies, and the need for standardization.

Independent of the technical concerns surrounding the collection and categorization of genetic material in fungal studies, there is also some ambiguity in the classifications of fungi. For instance, *Malassezia* are dimorphic, with both yeast and mycelial phases, and in the past had been placed in multiple genera [123]. The authors claim that, while the taxonomy for this particular genus has largely been resolved, older studies may miss this information, and this issue may also occur for other fungal organisms. Within the last decade, there was a push to end the system of dual nomenclature, as this approach came to be seen as archaic, and a single name classification has since begun to be adopted [126]. As fungal taxonomy continues to be expanded, NGS-based studies contribute greatly to the identification of new species, both with ITS-amplicon [74] and shotgun metagenomic techniques [127].

Complications in technical approaches and in classification have led to scarce investigations of the oral virome, but we can begin to draw a few conclusions from some of the recent work in this area. This is an important segment of microbiome research because not only can eukaryotic viruses affect the health of a host directly, but prokaryotic viruses can do so as well by altering the overall bacteriome composition and thus its function [75]. A study in Spain using single-virus genomics (SVG) and viral metagenomics in 15 saliva samples found 439 oral viruses, which they grouped into about 200 clusters that corresponded to genus-level classification [128]. They saw that most viruses were not consistently predominant, and it was difficult to define a core group of salivary viruses, and instead there were variable interpersonal compositions in the oral virome. However, 26 of their 200 viral clusters shared many genes, and most of these were *Streptococcus* phages, which is a reasonable finding since, as we have seen, *Streptococcus* is typically among the most abundant genera of oral bacteria, if not the most abundant, in Western oral microbiomes. Another study, also in Spain, of 72 healthy adult oral viromes had similar findings [129]. They found very few ubiquitous viruses while most were found only in individual samples, and once again *Streptococcus* phages were common. However, they did suggest a small core of oral viruses and pointed to the presence of viral cores in other body sites seen in other studies, including the lung in healthy samples [130], the gut even after fecal transplant [28], and the skin [29]. They also stress the specificity of this oral viral core to Western cultures, since virome research also suffers from the “WEIRD” bias mentioned above.

Protozoa and archaea are also components of the oral microbiome, though little has been said on either group. There do not appear to be any NGS-based explorations of oral protozoa, but instead they have been identified by microscopy techniques [30,131,132,133]. However, the presence of the 16S rRNA gene in archaea has led to the use of NGS techniques in some studies. All of the archaeal species thus far discovered in the oral cavity are methanogens (methane-producing organisms) of the phylum Euryarchaeota [134]. It has been shown that these archaea tend to be present at higher abundances in patients suffering from periodontitis [14,15]. However, it has been suggested that there may be more archaeal diversity that has as yet gone undetected, either because conventional methods have precluded the detection of other archaea, because they occur at low prevalence and abundance, or because of a lack of diversity in the populations sampled [135]. Each of these issues could be addressed with further explorations of NGS-based studies among diverse populations.

## 6. Oral Microbiome and Oral Diseases

The plant ecologist Robert Harding Whittaker, in defining terrestrial biomes in the 1970s, discussed gradients of environmental conditions ranging from favorable to extreme. He showed that both alpha and beta diversities decrease as biome conditions become more extreme. [136]. A parallel to this generalization has been seen with microbiome studies over the last decade if we consider that disease states equate to “extreme” environmental conditions within certain body sites. This frequently causes low alpha diversity (fewer distinct organisms), which leads to low beta diversity (uniqueness of an individual sample’s overall composition) as certain organisms become better equipped to dominate their habitat. Common diseases of the oral cavity, like periodontitis and dental caries, provide explicit examples of this phenomenon, wherein the microbiome composition is strongly tied to the disease state. However, at this stage in the development of the microbiome field, it is not always clear whether changes in microbial compositions lead to disease, or vice versa. Nevertheless, it is certainly worth discussing the associations that have been observed to begin to postulate microbiome-related mechanisms of disease origin or progression.

The species of the “red complex” (*Porphyromonas gingivalis*, *Treponema denticola*, and *Tannerella forsythia*) have historically been seen as the primary infective organisms implicated in periodontitis [137], but this was determined by culture-based studies, which thus missed much of the bacterial diversity present in samples. NGS techniques have since revealed other organisms that are also associated with periodontitis (Table 1, Figure 2), such as the classes Clostridia, Negativicutes, and Erysipelotrichia [16]; the genera *Synergistes* [17], *Prevotella*, and *Fusobacterium* [18]; and the species *Filifactor alocis* [16]; as well as the archaeal species *Methanobrevibacter oralis, Methanobacterium curvum/congolense*, and *Methanosarcina mazeii* [14,15]. Conversely, some organisms are associated with periodontal health, including the phylum Proteobacteria and the Firmicutes class Bacilli [16], and the genera *Streptococcus, Actinomyces,* and *Granulicatella* [19].

Clearly, there are many organisms associated in one way or another with periodontitis, but that raises the question of which may actually be causative agents, and which are merely impacted by environmental alterations in the disease state. One study that employed metatranscriptomics techniques compared the expression profiles of 160,000 genes and showed conserved differences in metabolism, despite variation in microbiome composition, suggesting that, in a disease state, the organisms present in a sample perform similar functions, even if species differ between samples [20]. This notion could be corroborated by another study, which proposed that methanogenic archaeal species develop syntrophic relationships by acting as “hydrogen sinks”, allowing for increased growth of pathogenic secondary fermenters. Members of the genus *Treponema* have a similar hydrogen-consuming activity, perhaps explaining their involvement in the “red complex”. Indeed, this study showed that abundances of *Treponema* and methanogenic archaea anti-correlated, suggesting that they may fill the same functional niche [15].

In dental caries, alpha diversity has been shown to diminish as the disease progresses and the species *Streptococcus mutans* has been found at high levels at early stages of caries development but not at later stages while other species of *Streptococcus* are associated with dental health [22]. It has been suggested that, while *S. mutans* is acidogenic and this may contribute to initial caries formation, other oral taxa are also acidogenic. The significant virulent factor in this situation is its ability to metabolize sucrose from a host’s diet into extracellular polysaccharides (EPS), which are necessary to produce cariogenic biofilms. Furthermore, adhesion between *S. mutans* and *Candida albicans* is promoted in this setting, with *C. albicans* providing additional acidogenesis [23].

Connections between the microbiome and cancer have also been explored recently. A number of species found in the oral cavity have been associated with oral cancer, including *Capnocytophaga gingivalis*, *Prevotella melaninogenica*, and *Streptococcus mitis* [24]. Table 1 lists a number of species that have been described as having associations with oral cancer, though there is the caveat that the samples in one of these studies were taken from tumor and non-tumor sites in the same patient, and thus the composition at both sites may in fact be affected by the disease, and it is unclear exactly what, if any, cancer-related treatment the patients have undergone [25]. Unfortunately, there do not appear to be many studies exploring this connection with modern techniques, nor is there much consensus among such studies on the microbiome composition in the presence of oral cancer, but there are a number of hypotheses on the potential cancer-promoting action of microbiota. It has been suggested that some of the normal oral bacteria, including *Streptococcus salivarius*, *Streptococcus intermedius*, and *Streptococcus mitis*, can convert ethanol to the carcinogen acetaldehyde [26,138] or upregulate cytokines and other proinflammatory molecules, leading to chronic inflammation that may be involved in carcinogenesis [139], and that bacterial toxins may also affect cell signaling pathways or damage DNA [140].

Esophageal cancer has also been explored, with associations seen with the periodontal pathogens *Tannerella forsythia* and *Porphyromonas gingivalis* (members of the “red complex”) [27]. The study showed that the genus *Neisseria* was linked to a lower risk of esophageal cancer, as was the carotenoid biosynthesis pathway, to which a number of *Neisseria* species can potentially contribute.

## 7. Oral Microbiome and Non-Oral Diseases

The microbiome of the oral cavity is by no means an isolated biome, but it is instead part of a highly interconnected series of microbiomes across the human body, forming a sort of micro-biosphere. As the entry point of nearly all ingested material, and due to its high vascularity, the oral cavity has ample opportunity to influence activity at other body sites. So, it is no surprise that, in addition to diseases of the oral cavity, the oral microbiome has been implicated in a number of systemic diseases.

The mouth is a direct route to both the lungs and the digestive system, so an association between oral taxa and disorders like cystic fibrosis (CF) [33] or colorectal cancer (CRC) [12,141,142,143] can perhaps be expected, given what we have already discussed. The pathogenicity of *Pseudomonas aeruginosa*, the primary agent in biofilm formation in the lungs of CF patients, can be inhibited by oral commensal streptococcal species, particularly *Streptococcus oralis*, through the production of hydrogen peroxide, which can disrupt biofilm production, but this was only observed if these streptococci were primary colonizers before the introduction of *P. aeruginosa*. However, in a typical CF lung environment, these streptococcal species actually stimulate the production of *P. aeruginosa* virulence factors, including elastase and pyocyanin [33].

Dysbiosis in the oral cavity resulting in periodontitis has been linked with oral, esophageal, gastric, lung, pancreatic, prostate, hematologic, and breast cancers [144,145], amongst others. Hypotheses for these connections include: Production of carcinogenic molecules like nitrosamines by nitrate-reducing taxa [146,147] or acetaldehyde by ethanol-metabolizing taxa [26,138], increased abundance of cancer-linked viruses like cytomegalovirus and Epstein–Barr virus [148,149], and, perhaps most prominently, increased proinflammatory markers stemming from immune reactions to periodontal disease like cytokines [139] and the receptor for advanced glycation end products (RAGE) [150].

Multiple studies have linked *Fusobacterium nucleatum* with CRC [141,142,143], as this is an oral commensal species that is highly invasive and adherent [151] and appears in adenoma samples of CRC patients. In adenoma cases, it was correlated with local inflammation, TNF-α, and IL-10 [141]. However, a recent NGS-based study found no association between *Fusobacterium* and CRC, but instead saw associations with the genera *Lactobacillus* and *Rothia* [12]. The authors suggest that the results of some other studies may actually be confounded by smoking, which has been shown to associate with *F. nucleatum* abundance in the mouth as well [152,153]. They also posit that, while *Lactobacillus* has been suggested as a probiotic when present in the gut microbiome [154,155], as seen in the previous section (see Table 1, Figure 2), this genus is associated with dental caries in the oral microbiome. Thus, *Lactobacillus* may not have a direct impact on colorectal carcinogenesis, but it could perhaps be an ancillary indicator of poor oral health, which we have seen is strongly linked to cancer.

Another study showed higher abundances of *Porphyromonas gingivalis* and *Aggregatibacter actinomycetemcomitans* in pancreatic cancer samples [31], both of which are keystone pathogens in periodontitis [18,21] As supporting evidence, they referenced a study that found that risk for pancreatic cancer was significantly increased in the presence of elevated serum antibodies to *P. gingivalis* [156], and another showing that both *P. gingivalis* and *A. actinomycetemcomitans* have the potential to initiate Toll-like receptor (TLR) pathways, which has been shown to be a driver of pancreatic carcinogenesis [157]. However, another study seems to contradict the first, finding a greater abundance of *Leptotrichia* in pancreatic cancer samples compared to healthy controls, and lower abundance of *Porphyromonas*, as well as lower abundance of *Aggregatibacter* (though the latter was not statistically significant) [32]. They suggest that a high ratio of *Leptotrichia* to *Porphyromonas* (LP ratio) is a biomarker for pancreatic cancer. In fact, they were able to reclassify one patient that was originally a healthy control but diagnosed with an unknown digestive disease. The individual’s high LP ratio prompted a re-evaluation that led to diagnosis of pancreatic cancer in that patient.

Combining the evidence presented in both studies may lead to a solution for the incongruous findings. Both reference the link between *P. gingivalis* antibodies and pancreatic cancer, so this may be due to the high abundance of this species prior to onset of the disease, leading to antibody production and eventual diminishing abundances. High initial *P. gingivalis* abundance would then potentially be linked to periodontal disease, which may then lead to various cancers as a result of systemic inflammation or any of the other mechanisms discussed here. Competition between *Leptotrichia* and *Porphyromonas* would explain their anti-correlation, so that as antibodies reduce the abundance of oral *P. gingivalis*, *Leptotrichia* is able to thrive. In fact, in the second study, some of the pancreatic cancer samples had low LP ratios, on par with non-pancreatic cancer LP ratios, so these may then have been early stage cases. This situation highlights a need for a greater focus on the temporal dynamics of the microbiome in these kinds of association studies to discover important factors across the onset, progression, and maintenance of disease states.

Some other systemic disorders are linked with periodontitis as well, like cardiovascular disease/atherosclerosis [34,35]. One study following over 3000 subjects for a 16-year period found that periodontitis with the loss of molars was linked with breast cancer (among other types), and premature death due to cancer and cardiovascular or gastrointestinal diseases [158]. As with the cancers that are linked to periodontitis, the resulting increase in systemic inflammation is a primary explanation for the link with cardiovascular disease [159]. This is largely due to the invasive nature of some of the associated taxa, like *P. gingivalis*, which also promotes invasiveness into host epithelial cells in species like *Prevotella intermedia*, an otherwise commensal oral species [160]. The proteins secreted by these organisms are implicated in their pathogenicity, such as gingipains from *P. gingivalis*, which aid in its biofilm formation during periodontitis [161], and subsequently activate cytokine production [162]. Studies linking periodontitis and atherosclerosis have relied on findings of oral bacteria colonizing atherosclerotic plaques [34,35] rather than on abundances of taxa in the oral cavity (Table 2, Figure 2). Although there appear to be fewer studies using NGS techniques of oral cavity samples to determine the associations between the oral microbiome composition and cardiovascular disease, the potential mechanisms of action by oral taxa make this another attractive area for investigation.

Rheumatoid arthritis (RA) has often been connected with periodontitis [36,163], once again implicating *P. gingivalis*, in this case for its production of gingipains and peptidylarginine deiminase, which enable protein citrullination, an important trigger for RA. However, recently it was shown that this direct connection may be erroneous [164]. In fact, studies of RA using 16S sequencing and whole metagenome shotgun sequencing, respectively, found that *P. gingivalis* was either not associated with RA [37] or actually more abundant in healthy controls compared to RA samples [38] while different organisms were associated with the disease (Table 2, Figure 2). This demonstrates how NGS studies can allow researchers to determine the veracity of previously held beliefs and potentially open new pathways for investigation.

Neurological disorders have also been associated with the oral microbiome. Perhaps the most complete study in this regard is the association of *P. gingivalis* with Alzheimer’s disease [40]. The authors of this study not only identified this bacterium in the brains of Alzheimer’s patients at levels that correlated with tau and ubiquitin aggregates (a hallmark of the disease), but also showed that *P. gingivalis* infection in mice resulted in brain colonization and increased the production of components of amyloid plaques. They went on to show that the gingipains proteases produced by *P. gingivalis* are neurotoxic and inhibit tau function. This suggests a direct connection between baring colonization of *P. gingivalis* and the origin or progression of Alzheimer’s disease and also suggests that gingipain inhibitors could be used to treat neurodegeneration in this disease. It has also been shown that typical oral species of the phylum Spirochaetes, including multiple species of the genus *Treponema*, often make up amyloid plaques, and that these organisms are capable of producing additional amyloid-β and amyloid-β protein precursor [41].

Dysbiosis of the oral microbiome is also implicated in disorders of the endocrine system. In this case, periodontitis appears to be a potential result of diabetes, as opposed to a potential cause as in most of the diseases discussed above. In one longitudinal study, the prevalence of periodontitis was 60% in subjects with diabetes and 36% in subjects without diabetes [165]. Some of the proposed mechanisms of the influence of diabetes on periodontal health include microangiopathy or alterations in the inflammatory response, collagen metabolism, or the glucose concentrations in gingival crevicular fluid [166]. Nevertheless, it has been proposed that periodontitis can also compound the effects of diabetes by upregulating the production of inflammatory factors like TNF-α, which can act as insulin antagonists [167]. Surprisingly, one NGS study showed that subjects with diabetes actually had lower abundances of the typical “red complex” species seen in periodontitis infections, *Porphyromonas gingivalis, Tannerella forsythia,* and *Treponema denticola* [43]. The authors note that potential confounders in their study include a higher mean plaque index and greater age in the subjects with diabetes. However, it is also possible that any of the proposed mechanisms of diabetic influence on periodontal health could result in a variation of the typical dysbiotic composition in periodontitis, or that the samples were collected at a stage in the progression of the disease in which the common pathogens are found at lower abundances. In either case, this presents another interesting opportunity for larger scale longitudinal studies, to discover potential alternative pathways to periodontal disease, and thus a greater comprehension of its mechanisms and potential treatments or preventative measures.

## 8. Clinical Potential of the Oral Microbiome/Manipulations and Perturbations of the Oral Microbiome

We have demonstrated here a few key examples of the wide-ranging action of the oral microbiome upon the human body. This opens vast possibilities for diagnosis and intervention, only some of which have begun to be explored, or even conceptualized. To fully leverage this potential, we will need to continue to probe the compositions and actions of oral microbiomes, both at small-scale interactions within individuals in different states of health, as well as at broad scales among different populations. The oral microbiome has already shown potential as a diagnostic tool. One machine learning-based study, which collected 2424 publicly available metagenomes from eight studies, showed that the performance of disease predictions was improved when using strain-level features (not feasible with 16S sequencing), and suggested that disease phenotypes are linked to “non-core” microbial genes/factors which may be found in variable genomic regions that are specific to strains/subspecies [90]. However, they caution the use of some potential biomarkers for diagnosing disease, such as the species *Streptococcus anginosus*, which actually associates with general dysbiosis rather than a particular disease. The authors still consider this work to be an early stage of modeling the healthy microbiome, so that it can be used to contrast states of dysbiosis associated with disease. Another attempt at using oral microbial abundances as biomarkers has shown some preliminary success in the early detection of colorectal cancer (CRC) lesions [92]. The fecal immune test (FIT) and the fecal occult blood test (FOBT) are typical non-invasive screening procedures used to detect CRC, but they suffer from poor sensitivity in detecting early lesions. The authors showed that a classification model based on oral microbiome samples had high specificity and greater sensitivity than standard tests in distinguishing CRC and polyp samples from healthy controls, with a further increase in sensitivity when combining with fecal microbiome samples. Thus, with further verification studies, the microbiome could be implemented to improve the rate of early detection of CRC.

In addition to a biomarker, the oral microbiome can be both a tool and a target for treating diseases. Commercially available probiotics, which contain live strains from bacterial genera, such as *Bifidobacterium*, *Lactobacillus*, and *Streptococcus*, have been shown to promote greater alpha diversity in the oral microbiome, though without making large-scale or permanent alterations to its composition [154]. As mentioned in the previous section, *Lactobacillus* in particular is a common and effective probiotic in the context of the gut microbiome, but it has been associated with dental caries, and indirectly with CRC. However, it is also possible that this effect is dependent upon the environmental context, as discussed above in the case of *Streptococcus oralis* during cystic fibrosis infection or *Leptotrichia* during pancreatic cancer. Thus, *Lactobacillus* without environmental conditions suitable for dental caries may instead help to promote greater alpha diversity and better oral health. This is another case of preliminary results with interesting potential that require deeper longitudinal study.

Mechanisms have been proposed for the beneficial impact of other potential probiotics as well, to counteract the progression of periodontal disease and caries, including strains of *Streptococcus salivarius*, which has been shown to downregulate inflammatory responses and to stimulate beneficial pathways like type I and II interferon responses [168]. Other potential probiotics for this use are *Streptococcus dentisani* [169] and *Streptococcus* A12 [170], which can buffer the acidic pH produced within cariogenic biofilms through arginine metabolism. The Proteobacteria species *Bdellovibrio bacteriovorus* has been suggested as a tool for potentially targeting periodontal pathogens [171]. It feeds on Gram-negative bacteria by invading them, eventually leading to lysis of its prey. Ex vivo experiments on saliva and subgingival plaque showed that *B. bacteriovorus* was able to attack two important oral pathogens, *Fusobacterium nucleatum* and *Aggregatibacter actinomycetemcomitans*, though not certain other desired targets, nor was it specific only to these targets.

Pathways and products of oral microbiota may also be treatment targets. The proinflammatory cytokine IL-17 was shown to be the most strongly upregulated in diabetic subjects and was associated with periodontitis while treatment with anti-IL-17 antibodies was able to mitigate this effect [172]. In Alzheimer’s disease, inhibitors of the gingipains proteases secreted by *Porphyromonas gingivalis* were able to reduce infection by this species in the brain as well as amyloid-β production and neuroinflammation [40].

There is still a lot to learn about the functionality of the oral microbiome, and its potential reaction to treatments. One instance stems from the “WEIRD” bias that has left many of the world’s populations out of this area of investigation. A study of “uncontacted” Amerindians in the Venezuelan Amazon, an isolated community with no known exposure to antibiotics, had bacteria carrying functional antibiotic resistance (AR) genes [45]. The authors suggest that “AR genes are likely poised for mobilization and enrichment upon exposure to pharmacological levels of antibiotics”. The authors emphasize the need for characterizing remote populations “before globalization of modern practices affects potentially beneficial bacteria harbored in the human body”. Thus, the context of microbiome studies must continue to be expanded before we can find the optimal approaches to treatment.

## 9. Conclusions and Future Outlook

Improvements in sequencing techniques and costs continue to propel the field of microbiome research forward, allowing for larger-scale approaches and wider understanding of its structure and function. However, over the last decade or so, much of the research has been exploratory and many investigators have taken their own approaches to perform experiments and analyses as the potentials and the limitations of these new techniques have been probed. Some amount of technical bias has undoubtedly permeated the results in this field as a result. As we begin to understand what NGS experiments show us, and as new approaches are developed, we must strive for standardized methods that will allow for reliable data comparison with minimal bias. The same is true for study designs and sample collection methods, as has been proposed [113]. The oral microbiome will benefit especially from concrete methodologies to ensure the proper context for a given study, because, depending on the goal of study, results may be dependent on particular niches within this habitat.

Current evidence allows for at least some tentative generalizations about the structure of the oral microbiome. Despite the arguments for the focus on gradients of abundances across samples of a population and against the reliance on stomatotypes (or other relevantly termed clusters of microbiome samples), stomatotypes allow for researchers to obtain a broad perspective and can help guide further analyses. The *Neisseria*/*Haemophilus* and *Prevotella*/*Veillonella* stomatotypes discussed above may represent relatively healthy compositions while the other stomatotypes that have been detected, which tend to be less consistent in terms of composition, may represent dysbiosis. The driver genera of the first two stomatotypes often appear to be associated with health while driver genera in other described stomatotypes are often associated with disease, like *Porphyromonas*, *Rothia*, and certain species of *Streptococcus*. Of course, there are exceptions, such as the associations that have been reported between periodontitis and *Prevotella* [18] or between dental caries and *Neisseria* [22], though to qualify that statement, as mentioned above, there may be alterations in the abundances of many taxa as a result of the disease state, not as a cause of the disease. So, as in the case of *Prevotella* in periodontitis or *Neisseria* in dental caries, these taxa may simply become opportunistic in the environment created by the disease (*Neisseria* may simply be taking advantage of the “hydrogen sink” created by archaeal methanogens and/or *Treponema* species, since its species tend to be acidogenic [173]), and therefore could still be considered to be associated with general oral health. Periodontitis and dental caries are heavily researched diseases, but in seeking associations between the microbiome and any disorder, it is important to thoroughly explore the mechanisms of disease progression before labelling any particular organism as a causative agent. Table 1 and Table 2 provided in this review, which list associated taxa, should be taken as just that: A list of potential associations to be further explored.

As has been emphasized throughout this review, our current conceptions of the oral microbiome are largely biased toward the lifestyles in “WEIRD” nations (Western, educated, industrialized, rich, and democratic) [46], as most of the investigators and studied samples come from these populations. The few studies referenced here that have incorporated non-Westernized samples highlight the holes in our current knowledge of the potential compositions and structures of the oral microbiome [44] and its functionality in particular contexts [45]. As researchers integrate more diverse populations into the field (as well as expanding the focus to include other domains of life present in the microbiome), they will be able to continually generate more extensive databases of human oral-associated taxonomy and functionality, allowing for more comprehensive studies.

Its position as the gateway to some of the most vital functions for human life tightly connects the oral cavity to the rest of the body and gives it a powerful influence on many of the body’s processes. Dysbiosis in the oral microbiome is clearly linked to a number of local and systemic diseases, though some of the particular associations and mechanisms of action remain conjectural pending further study. Some of the treatment examples discussed here appear straightforward, even despite the need for deeper verification of efficacy. However, the human microbiome is a multi-dimensional interconnected micro-biosphere, and perturbations may have as yet unforeseen effects, whether undetected in the short term or undeterminable in the long term without the appropriate study designs. We have already mentioned examples where unrelated organisms can fill some of the same functional niches (see the discussion above of *Treponema* and methanogenic archaeal species acting as hydrogen sinks in periodontitis) and their competition can be implicated in disease. Microbiome research is far from complete, and without a deep understanding of its nature, we should exercise caution and patience before widely exploiting its medical potential. This will require many more large-scale and longitudinal studies, greater focus on the functional component of the microbiome, and a stronger characterization of the varying structures of each microbiome of the body in different contexts, part of which will be to combat the WEIRD bias, which has thus far limited the scope of study, and the dearth of attention to the less prominent domains of the microbiome, like fungi, archaea, and viruses.

## Figures and Tables

**Table 1:** Examples of metagenomic studies of associations between the oral microbiome and oral diseases. The first column indicates a disease, the second indicates organisms that have been found at higher abundances in individuals presenting with the disease, the third indicates organisms at lower abundances, and the fourth contains the references to the literature, which displays these findings. (*) indicates taxa associated with oral cancer from a study in which samples were from tumor and non-tumor sites in the same patients and disease treatment is not specified.

Disease	Associated Organisms	Inhibited Organisms	Reference
Periodontitis	Phyla: Spirochaetes, Synergistetes and Bacteroidetes Classes: Clostridia, Negativicutes and Erysipelotrichia Genera: *Prevotella, Fusobacterium* Species: *Porphyromonas gingivalis*, *Treponema denticola, Tannerella forsythia, Filifactor alocis, Parvimonas micra, Aggregatibacter actinomycetemcomitans* Archaea: *Methanobrevibacter oralis, Methanobacterium curvum/congolense*, and *Methanosarcina mazeii*	Phyla: Proteobacteria Classes: Bacilli Genera: *Streptococcus, Actinomyces, Granulicatella*	[14,15,16,17,18,19,20,21]
Dental caries	Genera: *Neisseria, Selenomonas, Propionibacterium* Species: *Streptococcus mutans, Lactobacillus* spp. Fungi: *Candida albicans*	Species: non-mutans *Streptococci, Corynebacterium matruchotii, Capnocytophaga gingivalis, Eubacterium IR009, Campylobacter rectus, Lachnospiraceae sp. C1*	[22,23]
Oral cancer	Species: *Capnocytophaga gingivalis*, *Prevotella melaninogenica* and Streptococcus mitis, Peptostreptococcus stomatis*, Streptococcus salivarius*, Streptococcus gordonii*, Gemella haemolysans*, Gemella morbillorum*, Johnsonella ignava* and Streptococcus parasanguinis I*	Species: Granulicatella adiacens*	[24,25,26]
Esophageal cancer	Species: *Tannerella forsythia, Porphyromonas gingivalis*	Genera: *Neisseria* Species: *Streptococcus pneumoniae*	[27]

**Table 2:** Examples of metagenomic studies of associations between the oral microbiome and systemic diseases. The first column indicates a disease, the second indicates organisms that have been found at higher abundances in individuals presenting with the disease, the third indicates organisms at lower abundances, and the fourth contains the references to literature which displays these findings.

Disease	Associated Organisms	Inhibited Organisms	Reference
Colorectal cancer	Genera: *Lactobacillus*, *Rothia* Species: *Fusobacterium nucleatum*		[12,28,29,30]
Pancreatic cancer	Genera: *Leptotrichia* (later in progression of disease) Species: *Porphyromonas gingivalis and Aggregatibacter actinomycetemcomitans* (at onset of disease)	Genera: *Leptotrichia* (at onset of disease) Species: *Porphyromonas gingivalis and Aggregatibacter actinomycetemcomitans* (later in progression of disease)	[31,32]
Cystic fibrosis	Species: *Streptococcus oralis* (depends on environmental conditions), S. *mitis*, *S*. *gordonii* and *S*. *sanguinis*	Species: *Streptococcus oralis* (depends on environmental conditions)	[33]
Cardiovascular disease	Species: *Campylobacter rectus*, *Porphyromonas gingivalis*, *Porphyromonas endodontalis*, *Prevotella intermedia*, *Prevotella nigrescens,* (oral commensals that were found on athersclerotic plaques - not necessarily at high abundance in oral cavity)		[34,35]
Rheumatoid arthritis	Genera: *Veillonella, Atopobium, Prevotella, Leptotrichia* Species: *Rothia mucilaginosa, Rothia dentocariosa, Lactobacillus salivarius, Cryptobacterium curtum*	Genera: *Haemophilus, Neisseria* Species: *Porphyromonas gingivalis, Rothia aeria*	[36,37,38,39]
Alzheimer’s disease	Phyla: Spirochaetes Species: *Porphyromonas gingivalis*		[40,41,42]
Diabetes	Genera: *Aggregatibacter, Neisseria, Gemella, Eikenella, Selenomonas, Actinomyces, Capnocytophaga, Fusobacterium, Veillonella, Streptococcus*	Genera: *Porphyromonas, Filifactor, Eubacterium, Synergistetes, Tannerella, Treponema*	[43]

**Table 3:** Consensus stomatotype-driving genera. Genera that have been shown in the literature to strongly drive the distinction between samples of oral microbiome datasets by differences in their abundances. The stomatotype numbers are arbitrarily assigned. The genera are listed in the second column, along with notes on associations between the organisms where relevant. The third column contains the references to the literature, which shows these stomatotype associations.

	Genus	References
Stomatotype 1		
	***Neisseria***	[10,48,49,51]
	***Haemophilus***	[10,48,51]
Stomatotype 2		
	***Prevotella***	[10,48,49,51]
	***Veillonella***	[10,48,51]
Variable Stomatotypes		
	***Streptococcus***—varies depending on study and species	[10,48,49,51]
	***Gemella***—co-occurs with *Streptococcus* and *Porphyromonas*	[49,51]
	***Porphyromonas***—may co-occur with *Streptococcus, Gemella*, or *Neisseria*	[48,51]
	***Rothia***—co-occurs with varying species of *Streptococcu*s, depending on study	[49,51]
